# Solvent-Free Preparation of Tannic Acid Carbon Dots for Selective Detection of Ni^2+^ in the Environment

**DOI:** 10.3390/ijms23126681

**Published:** 2022-06-15

**Authors:** Yan Li, Can Liu, Menglin Chen, Yulong An, Yunwu Zheng, Hao Tian, Rui Shi, Xiahong He, Xu Lin

**Affiliations:** 1 Yunnan Key Laboratory of Wood Adhesives and Glued Products National Joint Engineering Research Center for Highly-Efficient Utilization of Forest Biomass Resources, Southwest Forestry University, 300 Bailong Road, Kunming 650224, China; ly97111659@163.com; 2 Key Laboratory for Forest Resources Conservation and Utilization in the Southwest Mountains of China, Ministry of Education, Southwest Forestry University, Kunming 650224, China; shirui@swfu.edu.cn (R.S.); hexiahong@hotmail.com (X.H.); 3 National Joint Engineering Research Center for Highly-Efficient Utilization Technology of Forestry Resources; Southwest Forestry University, Kunming 650224, China; CML13529707162@163.com (M.C.); ayl96yu@163.com (Y.A.); zyw85114@163.com (Y.Z.); 4 Agro-Products Processing Research Institute, Yunnan Academy of Agricultural Sciences, Kunming 650000, China; tianhao.630@163.com

**Keywords:** biomass carbon dots, solvent-free, tannic acid, Ni^2+^ detection

## Abstract

Carbon dots (CDs) are widely used nanomaterials that not only exhibit good biocompatibility and photostability, but also benefit from a simple preparation process and easy functionalization, making them promising for broad applications in the fields of heavy metal ion detection and optoelectronic devices. Based on the excellent optical properties of CDs and the current situation of increasing energy shortages, this paper selects the natural polyphenolic compound tannic acid (TA) found in biomass materials as the carbon source and innovatively adopts a simple and convenient solvent-free pyrolysis method without auxiliary reagents or solvents. The CDs with good water solubility and certain fluorescence properties were directly prepared under the condition of high temperature, and the obtained CDs exhibited blue fluorescence, and a high QY of 35.4% was obtained at 300 °C. The analysis and results demonstrate the selectivity of these CDs for the detection of various metal ion solutions. In particular, these CDs are sensitive to Ni^2+^ and can be used as fluorescent sensors for the efficient and sustainable detection of Ni^2+^, whereas previous sensors were often specific to Fe^3+^ and Hg^2+^. Thus, a new sensing technique has been developed for the detection of Ni^2+^ to achieve more sensitive and rapid detection.

## 1. Introduction

Heavy metal pollution has attracted much attention because of its harmful effects on the environment and human health. Many metal ions have high chemical toxicity and can migrate to, and accumulate in, the human body through the atmosphere, water and soil environment, and through the food chain, which may lead to harmful effects such as irreversible mutation, oxidative stress and serious damage to the central nervous system [1,2]. Therefore, it is very important to detect and quantify these ions efficiently, sensitively and quickly by environmental monitoring. Several methods have been developed for the detection of heavy metals in water, including atomic absorption spectrometry [3], atomic emission spectrometry [4], X-ray fluorescence spectrometry [5], mass spectrometry [6], UV–vis spectroscopy [7], electrochemical methods [8], and laser-induced breakdown spectrometry [9,10]. These techniques have a good detection range and have been proven to provide accurate results; however, they have some shortcomings, such as expensive instruments, relatively complex operation, inconvenient execution, and the difficult analysis of a large number of samples. A more practical method of environmental pollutant sensing involves the use of electrochemical analysis (i.e., electrochemical sensors) [11,12]. Although these methods are used more generally for detecting analytes and enabling measurements in the field, they require the use of sensitive electronic components under carrier gas flow. Therefore, it is necessary to design a fast, sensitive and inexpensive method to detect these ions. To date, many fluorescence and plasma nanomaterial analysis methods dependent on semiconductor quantum dots and organic molecules have been developed to quantitatively detect heavy metals in aqueous media by monitoring the change in signal intensity [13,14]. However, their application in this field may be hampered by the challenges associated with synthetic procedures and the need for expensive or sometimes toxic reagents. On the other hand, carbon dots (CDs) can provide an interesting and multifunctional alternative to currently available systems. In addition, nickel is a relatively abundant trace element and is widely used in industrial production, so Ni^2+^ is found in three kinds of wastes. When smelting nickel ore and smelting steel, part of the ore powder enters the atmosphere with the airflow, and nickel and its compounds are also discharged during the roasting process, eventually becoming particulate matter in the atmosphere. When the nickel dust generated by combustion encounters hot carbon monoxide, it generates volatile and highly toxic carcinogens. Wastewater from the nickel-plating industry, machine-manufacturing industry and metal-processing industry often contains nickel, which causes nickel enrichment in soil, making Ni^2+^ abundant in nature and able to enter the human body in various ways. However, the harmful effect of Ni^2+^ in the human body is extremely serious. Contact with volatile nickel ions causes contact dermatitis and even skin erosion. Long-term chronic exposure to nickel also causes gastrointestinal bleeding, hair loss, etc. A large amount of exposure to nickel ions can cause irreversible damage, such as chronic renal insufficiency and liver insufficiency. Therefore, the detection of Ni^2+^ is of great significance.

There have been a number of recent reports on potentiometric Ni^2+^ selective electrodes in the literature; however, most of these electrodes possess either one, two or, in some cases, all of the following problems: (1) high limit of detection, (2) narrow working concentration range and (3) serious interferences from various cations, which seriously limit their application. There are also some studies showing good selectivity for the detection of Ni^2+^: polymers as coating materials were combined with quartz crystal microbalances (QCMs) to design sensor devices for the detection of both ionic and neutral analytes in liquid phase [15]; Shamsipur et al. [16] prepared a highly sensitive and highly selective fluorescent photoelectrode membrane for ultra-trace Ni^2+^ ion determination, which was successfully applied to the determination of trace Ni^2+^ ions in edible oil and electroplating nickel industrial wastewater samples; and the use of pentacyclooctaaza as a neutral carrier in the construction of a new PVC membrane electrode selective to Ni^2+^ ions was successfully applied to the determination of nickel content of chocolate and milk powder samples, although there was a limit to the pH of the solution [17].

CDs constitute a new type of fluorescent carbon nanoparticles whose particle size is usually in the range of 1–10 nm [18]. CDs were discovered during the purification of carbon nanotubes by Xu et al. [19] in 2004 and named by Sun et al. [20] in 2006, and these materials have been shown to have the advantages of good water dispersion, nontoxicity, biocompatibility, polychromatic fluorescence, fluorescence stability, easy functionalization, easy preparation, environmental friendliness, low cost, and so on [21]. CDs have very broad application prospects in the fields of electrochemical detectors [22], biosensors [23], fluorescent probes [24], biological imaging [25], cell labeling [14], nanodrugs [26], photocatalysts [27,28], drug carriers and controlled release materials [20]. In particular, the interaction between CDs and metal ions will cause changes in the fluorescence signal of the CDs, so the CDs can be used as fluorescent probes [29,30] to detect metal ions quickly and sensitively, replacing the traditional expensive and difficult detection methods. Chen et al. synthesized nitrogen-doped carbon dots (N-C dots) by one-pot solid-phase pyrolysis of citric acid and guanidine hydrochloride, and these N-C dots can be used as on–off fluorescence probes for the detection of Fe^3+^. The detection mechanism involves dynamic and static fluorescence quenching [31]. Liu et al. synthesized fluorescent CDs by a one-step hydrothermal method using chocolate as a raw material, and these CDs can be used to sensitively and selectively sense Pb^2+^. The detection range was from 0.033 to 1.67 μM, and the detection limit was as low as 12.7 μM [32]. Due to the simplicity and sensitivity of their preparation process, CDs have been gradually used to detect metal ion contents inside and outside organisms [33,34].

In recent years, the preparation of CDs has received increasing attention, and the raw materials and synthetic methods have become the focus of research [35]. Compared with traditional toxic and expensive synthetic materials, biomass materials for the preparation of CDs have attracted increasing attention from researchers in today’s increasingly tense energy environment. Compared with other carbon sources, biomass carbon sources are eco-friendly and natural without additives, and they have many advantages for the preparation of CDs, including low price, easy access, environmentally green nature and high abundance. Therefore, the selection of an appropriate carbon source and the design and synthesis of multifunctional composite materials are very important. Natural polyphenolic compounds with the characteristics of abundant surface groups and easy functionalization have become excellent biomass raw materials for the preparation of CDs. TA has a central glucose unit with 10 peripheral gallic acids and 25 phenolic hydroxyl groups, which are easy to hydrolyze and can participate in various reactions, so it has become the first choice for the preparation of CDs.

Among synthesis methods, the common solvothermal synthesis of CDs involves expensive or toxic raw materials and solvents, as well as complex processes, harsh synthesis conditions and high costs, which limit the wide application of this method. Therefore, the search for green raw materials and efficient preparation methods has become a research hotspot. In terms of preparation methods, the solvent-free pyrolysis method is a pioneering new strategy to obtain CDs. Han et al. [36] proposed a direct one-step physical method to extract highly fluorescent carbon quantum dots from carbon black. Compared with the traditional synthetic methods to prepare biomass CDs, this method uses only renewable natural products as raw materials without any additional solvents or catalysts, making the whole experimental process simple and green, and, due to the diversity of raw materials, CDs with different properties and structures can be obtained.

**Figure 1:**
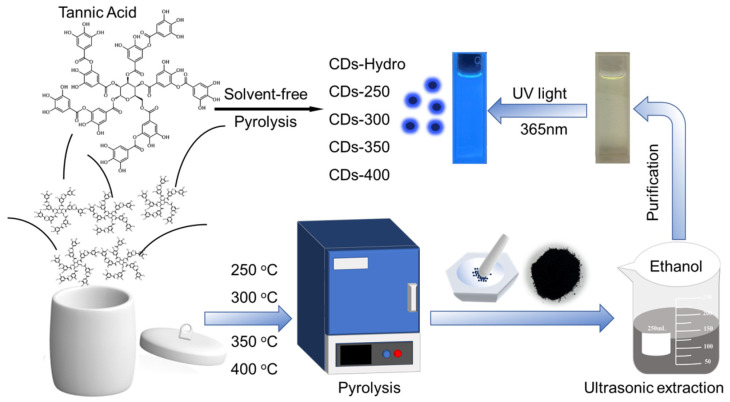
The synthesis route of TA-based CDs by solvent-free pyrolysis and basic preparation flow chart.

Herein, we report a method for the preparation of fluorescent CDs by solvent-free pyrolysis using natural polyphenols (TA). Since biomass char is not completely carbonized or burned to ashes when carbonized at different temperatures, the temperature range used for TA is 250–400 °C, with 50 °C as the temperature gradient. After the biomass carbon black was obtained, it was added to ethanol and extracted for a period of time. After purification, a CD solution with bright blue fluorescence was obtained under ultraviolet light excitation at 365 nm (Figure 1). According to the different pyrolysis temperatures, the obtained CDs were named CDs-250~CDs-400. To distinguish them from the CDs prepared by the traditional hydrothermal method, the CDs prepared by the hydrothermal method using TA as a precursor were named CDs-Hydro. No blue fluorescence was observed when the pyrolysis temperature of TA was below 250 °C or above 400 °C. Most importantly, we further demonstrated that these CDs can serve as very efficient fluorescent sensing platforms for the label-free, sensitive and selective detection of Ni^2+^, with a detection limit as low as 20 µM, and they were successfully applied to the detection of Ni^2+^ in actual water samples. Compared with the methods used in other studies, this method has the advantages of an easily available carbon source, low cost, and simple experimental performance, which is of great significance for the large-scale industrial production and wide application of CDs.

## 2. Results

### 2.1. TGA-MS Analysis of Raw Materials

**Figure 2:**
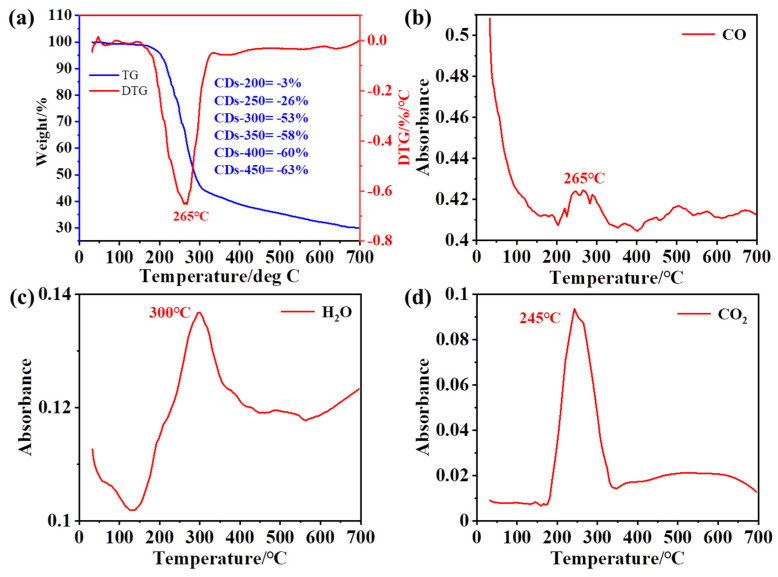
(**a**) TG/DTG curve of tannic acid. (**b**–**d**) The evolution of gaseous products for the components at 10 °C min^−1^.

The pyrolysis reaction of TA was monitored by thermogravimetric analysis and mass spectrometry (TGA-MS) (Figure 2). The main weight loss process during biomass pyrolysis is generally divided into two stages: (I) decomposition of unstable compounds and (II) continuous devolatilization [37]. As shown in Figure 2a, the main weight loss range of TA was 200–400 °C, and the initial decomposition temperature was 250 °C. The decomposition temperature range is wide, and approximately 30% of the solid residue was ultimately retained. The mass loss of the sample was approximately 1% due to the release of TA volatiles below 200 °C. TA was pyrolyzed from 200 °C to 340 °C. At this stage, the sample was mainly depolymerized, and most of the samples were decomposed into volatile components [38], which produced CO, CO_2_, H_2_O and other compounds after the decomposition reaction [39], with the main degradation occurring at approximately 265 °C. The loss of the sample was slow between 340 °C and 700 °C and finally reached a high carbon formation rate of 30%. The carbonization process reforms the polycyclic aromatic structures [40,41]. During pyrolysis, the sample was decomposed into small volatile components. In this experiment, the volatiles were analyzed by real-time mass spectrometry, and three main small-molecule gas products were detected at *m*/*z* = 18 (H_2_O), *m*/*z* = 28 (CO) and *m*/*z* = 44 (CO_2_) (m represents the number of protons, and z represents the charge). As shown in Figure 2, the above three small molecules were eliminated at approximately 250–300 °C in TA, which is consistent with the temperature at which the maximum weight loss rate occurred. Notably, the maximum elimination rate of water occurred at 300 °C. This result, combined with the later quantum yield data, means that the formation of carbon dots is closely related to the elimination of water.

### 2.2. Optical Properties

**Figure 3:**
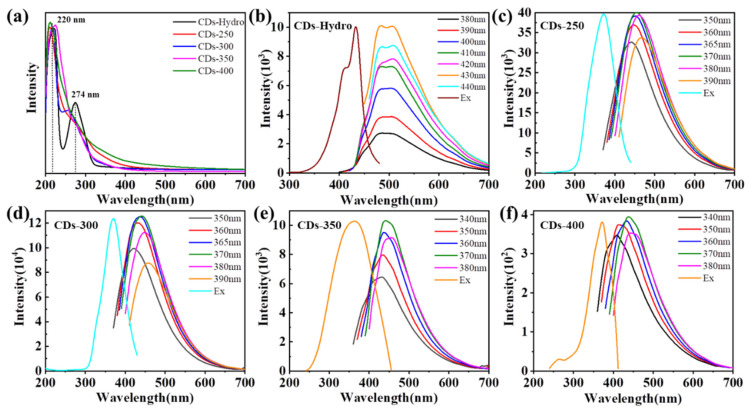
(**a**) UV/vis absorption spectra of CDs based on TA in ethanol solution. (**b**) PL emission spectra and PL excitation of CDs-Hydro in ethanol solution. (**c**–**f**) PL emission spectra and PL excitation of CDs-250 to CDs-400 in ethanol solution.

The UV/Vis absorption spectra of the CDs in ethanol were measured, as shown in Figure 3a. The CDs prepared by the two synthesis methods gave rise to fluorescence absorption peaks at 220 nm, corresponding to the π-π*transition of the C=C bond in the carbon nucleus [42]. The CDs-Hydro prepared by the hydrothermal method gave rise to an obvious absorption peak at 274 nm, and CDs-300 and CDs-350 prepared by the solvent-free pyrolysis method gave rise to absorption peaks at 256 nm and 276 nm, respectively, which can be attributed to the eigenstate (π-π*) transition of the aromatic sp^2^ domain in the carbon nucleus. Unlike many other reported CDs, our CDs did not exhibit surface defect state absorption in the visible region. Figure 3b–f show the fluorescence emission (PL) and excitation spectra of five CDs in ethanol solutions. The maximum excitation wavelength from CDs-250 to CDs-400 was 370 nm, and the maximum emission peak was approximately λ = 450 nm, corresponding to blue fluorescence. In contrast, the best excitation wavelength of CDs-Hydro was 430 nm, and the maximum emission peak was at approximately λ = 490 nm–500 nm, corresponding to blue–green fluorescence. The emission of the CDs was almost independent of the excitation wavelength [42].

**Figure 4:**
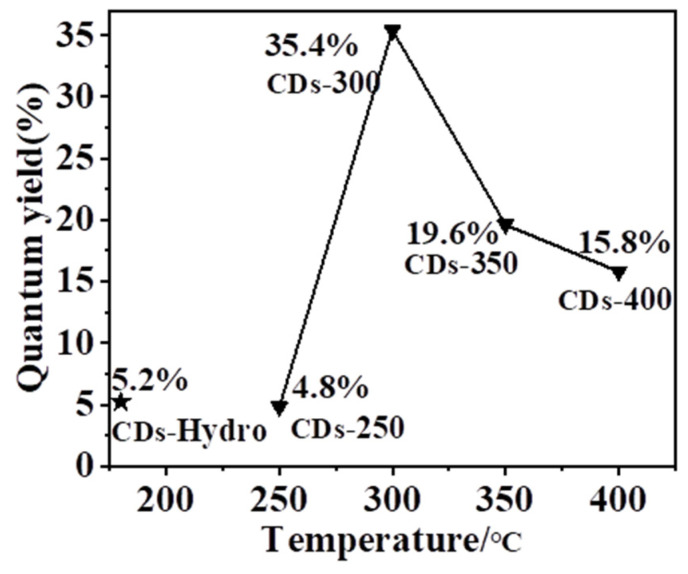
Dotted line plot of CDs quantum yield versus reaction temperature.

Different carbonization temperatures had no significant effect on the maximum emission peak of the CDs but did have a significant effect on the fluorescence quantum yield (QY) (Figure 4). With an increase in the carbonization temperature, the QYs of these CDs first increased and then decreased, reaching a maximum value of 35.4% at 300 °C, which was seven times the QY of the control CDs-Hydro, reflecting the high efficiency of the CDs prepared by a solvent-free pyrolysis method. In conclusion, the QY of biomass-based CDs prepared by the solvent-free pyrolysis method was better than that of biomass-based CDs prepared by the traditional solvothermal method.

### 2.3. Surface Morphology and Characterizations

**Figure 5:**
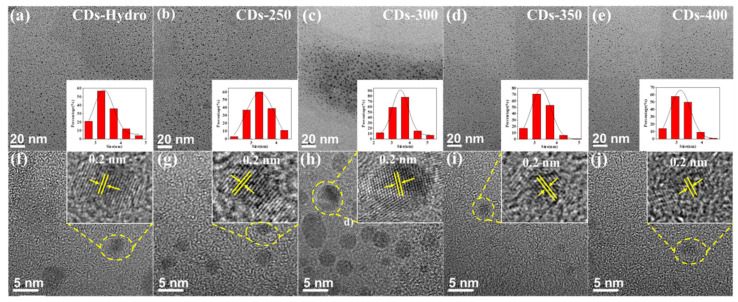
(**a**–**e**) TEM images of CDs-Hydro, CDs-250 to CDs-400, inset: histograms and Gaussian fittings of particle size distribution. (**f**–**j**) HR-TEM images of CDs-Hydro, CDs-250 to CDs-400.

The morphology of the prepared samples was analyzed by transmission electron microscopy (TEM). As shown in Figure 5a–e, the TEM images show that the samples were well dispersed and that all the materials formed dots with similar average particle sizes. The average particle sizes of the control CDs-Hydro and the solvent-free CDs-250 to CDs-400 were 3.3, 3.4, 3.5, 3.3 and 3.2 nm, respectively. With increasing carbonization temperature, the particle size changed less, and the quantum size effect was not the dominant mechanism responsible for the chromatic emissions. The high-resolution TEM images (Figure 5f–j) of the samples show that graphite carbon has lattice stripes with good resolution, and the crystal plane spacing was found to be 0.2 nm, which is similar to the (100) crystal plane of graphite carbon.

**Figure 6:**
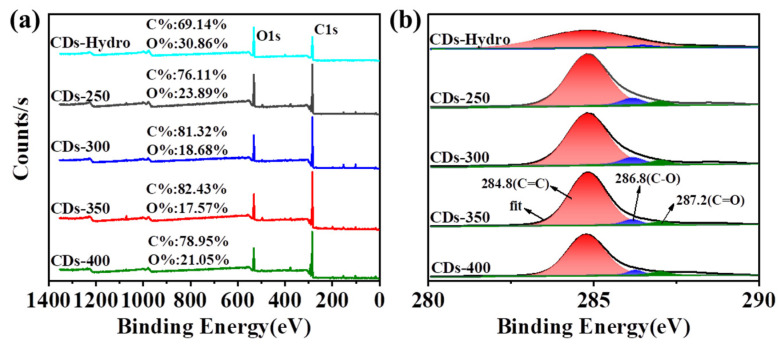
(**a**) XPS survey spectra, and (**b**) high-resolution C1s spectra of CDs-Hydro and CDs-250-400.

The functional groups of the five CD samples were studied by X-ray photoelectron spectroscopy (XPS) to determine the group distribution characteristics of the obtained CDs (Figure 6). The whole XPS spectrum clearly shows the ionization peaks of C1s (284.7 eV) and O1s (532.4 eV) (Figure 6a). In conclusion, the hydrothermally prepared CDs exhibited a lower degree of graphitization and lower fluorescence QY compared to the CDs formed by the solvent-free pyrolysis method. In addition, to further study the surface states of the CDs, the peak area was analyzed. As shown in Table 1, the resolved C1s XPS spectra show peaks at 284.8, 286.8 and 287.2 eV (Figure 6b), indicating the presence of C=C/C-C (sp^2^ carbon), C-O (sp^3^ carbon) and C=O (carbonyl carbon), respectively. For the CDs prepared by the solvent-free pyrolysis method, with increasing carbonization temperature, the sp^2^ carbon content first increased and then decreased, indicating that high temperatures induce the oxidative degradation of tannic acid. Detailed analysis showed that the sp^2^ carbon content in CDs-300 was the highest, and this was the primary factor leading to the high fluorescence efficiency. When the pyrolysis temperature exceeds 300 °C, the excessive oxidation leads to the decrease in the graphite degree and the fluorescence QY.

### 2.4. The Fluorescence Quenching of CDs by Ni^2+^

**Figure 7:**
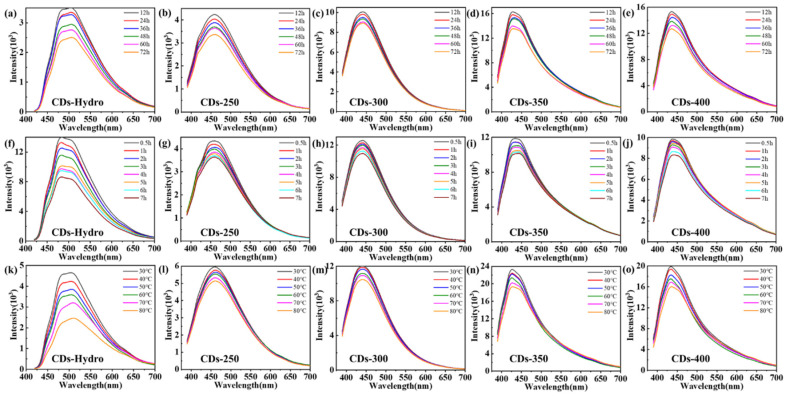
(**a**–**e**) PL emission spectra of CDs-Hydro, CDs-250-400 at different durations under visible light in ethanol solution. (**f**–**j**) PL emission spectra of CDs-Hydro, CDs-250-400 at different durations under ultraviolet light in ethanol solution. (**k**–**o**) PL emission spectra of CDs-Hydro, CDs-250-400 in a water bath at different temperatures in ethanol solution.

**Figure 8:**
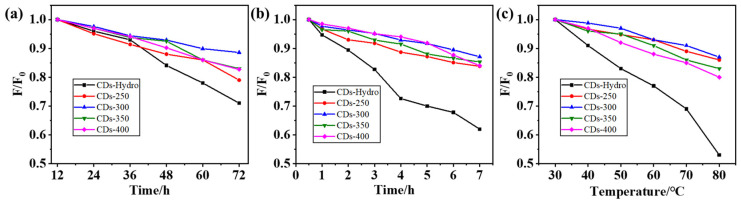
Decay curve of PL intensity of CDs-Hydro, CDs-250-400 with increasing (**a**) visible and (**b**) UV irradiation time. (**c**) Decay curve of PL intensity of CDs-Hydro, CDs-250-400 with increasing temperature.

**Figure 9:**
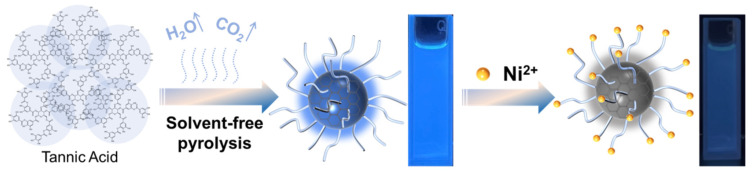
Schematic diagram of the mechanism of TA-based CDs synthesized by solvent-free pyrolysis for the detection of Ni^2+^.

**Figure 10:**
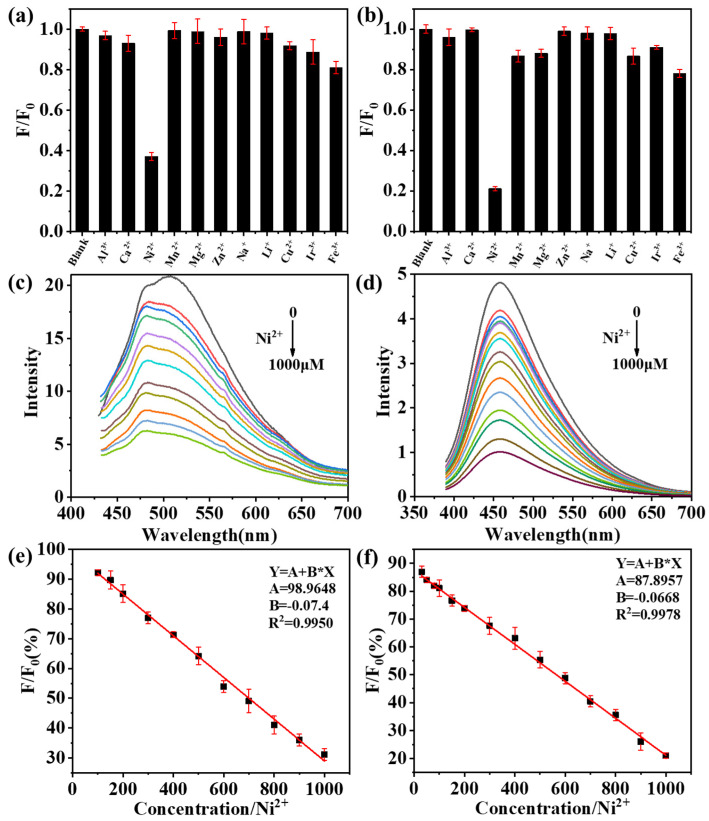
(**a**,**b**) Selectivity of CDs-Hydro and CDs-300 to different metal ions, F_0_ and F present the fluorescence intensity of CDs before and after adding metal ions. (**c**,**d**) Fluorescence emission spectra of CDs-Hydro and CDs-300 after the addition of different concentrations of Ni^2+^. (**e**,**f**) Linear relationship between fluorescence intensity ratio of CDs-Hydro and CDs-300 solution and Ni^2+^ concentration at different Ni^2+^ concentrations.

CDs can combine with some groups, such as sulfhydryl and carboxyl groups, on the surface of heavy metal ions to induce fluorescence quenching. Because the CDs obtained by the solvent-free pyrolysis method have excellent light stability and thermal stability (Figure 7 and Figure 8), these CDs are promising candidates for the detection of Ni^2+^ in the environment. In addition, the surface of CDs can be modified by specific groups (such as carboxyl, amino and hydroxyl groups), which improves their selectivity to targets, so these CDs can be used as detection probes for specific ions(Figure 9). We selected eleven metal ions (Al^3+^, Ca^2+^, Ni^2+^, Mn^2+^, Zn^2+^, Mg^2+^, Na^+^, Li^+^, Cu^2+^, Ir^3+^, and Fe^3+^) as the detection objects. Figure 10 shows that the two CDs were selective to different metal ions, with the response to Ni^2+^ being the most significant. We believe that, due to the change in the electronic structure of the CDs, the surface hydroxyl groups interacted with Ni^2+^, resulting in fluorescence quenching. Therefore, to further study the sensing response of these two kinds of CDs to Ni^2+^, we prepared different concentrations of Ni^2+^ (0–1000 μM) and evaluated their effects on the fluorescence intensity of CDs. The fluorescence intensity of the CDs varied linearly with the Ni^2+^ concentration, with a correlation coefficient of R^2^ ≥ 0.996. In conclusion, the CDs obtained in this study exhibited good selectivity for Ni^2+^, supporting the use of these CDs as metal-ion-detection probes. The detection limit of the CDs obtained by the solvent-free pyrolysis method was less than 20 µM, while the detection limit of the control CDs was only 100 µM, indicating that the CDs obtained by the solvent-free pyrolysis method are more sensitive for the detection of Ni^2+^.

## 3. Discussion

In general, there are two common CD PL mechanisms. One is the band gap transition based on the conjugate structure of the sp^2^ carbon core, and the other is related to the surface defects of CDs. Because the absorption spectra of our samples did not indicate the presence of surface defects, we believe that the band gap transition of the conjugated structure was the main factor controlling the PL in our system. This speculation about the fluorescence mechanism is also consistent with the experimental XPS results. With increasing reaction temperature, TA condenses together through dehydration and the removal of hydroxyl and other functional groups by carbon dioxide to form a conjugated graphite carbon structure, and then emits fluorescence through electronic transitions. According to the experimental results, the CDs prepared by solvent-free pyrolysis have a better graphitization degree and better fluorescence QY than those prepared by the hydrothermal method.

In summary, we prepared CDs with high QYs and stable blue fluorescence using the simplest and most environmentally friendly solvent-free pyrolysis method with TA as a starting material. The synthesis temperature affected the QY and surface states of the CDs. The QY value of CDs-300 was the highest, reaching 35.4%, and the higher C=C/C-C content of CDs-300 indicates that CDs can achieve a higher degree of graphitization at this temperature. The surface of the CDs was rich in oxygen-containing functional groups, which are more conducive to the coordination of Ni^2+^, resulting in high selectivity towards Ni^2+^, with good linearity, and these CDs are thus suitable as fluorescent Ni^2+^ sensors. The CD preparation method in this study is simple, green and novel; the reaction solvent can be recycled, which reduces the production cost of the CDs; and new suggestions for the development of biomass materials are provided. Previous methods were simpler and less expensive. In conclusion, this study provides a new idea for the synthesis of CDs and the detection of metal ions.

## 4. Materials and Methods

### 4.1. Materials

Tannic acid (99.0%), ethanol (99.7%), FeCl_3_ (99.0%), AlCl_3_ (99.0%), CaCl_2_ (99.0%), MgCl_2_ (99.0%), CuCl_2_ (99.0%), ZnCl_2_ (99.0%), NiCl_2_ (99.0%), MnCl_2_ (99.0%), LiCl (99.0%), and IrCl_3_ (99.0%) were provided by Shanghai Titan Science Co., Ltd (Shanghai, China). Unless otherwise stated, all reagents were used as is and no further purification was required.

### 4.2. Methods

The sample was pyrolyzed by using the model (Japanese Science, thermo plus EV2/ thermo mass photo) Thermogravimetry-Mass Spectrometer (TG-MS) to analyze the experiment. Transmission electron microscopy (TEM) was carried out using an FEI Tecani G2 F20 operating at an acceleration voltage of 200 kV. UV-vis spectra were recorded with a Shimadzu UV-2600 spectrometer. Fluorescence measurements were collected using a Shimadzu fluorescence spectrophotometer RF-6000. QYs of the obtained three CDs were determined by a relative method. X-ray photoelectron spectroscopy (XPS) was investigated by using K-Alpha spectrometer with a mono X-Ray source Al Kα excitation (1486.6 eV). Binding energy calibration was based on C1s at 284.7 eV. HORIBA Scientific LabRAM HR Evolutio was used for Raman analysis. QYs of the obtained three CDs were determined by a relative method. Specially, quinine sulfate (QY = 55% in 0.1 M H2SO4) was selected as the reference for the blue emission, rhodamine 6G (QY = 95% in ethanol) for the green emission, and rhodamine B (QY = 56% in ethanol) for the red emission.

### 4.3. Synthesis of CDs

First, 1.0 g of tannic acid was placed in separate 50 mL crucibles, heated in a muffle furnace at different temperatures for 6 h, and then cooled naturally to room temperature. The biomass-derived carbon black was ground and placed in a beaker containing 100 mL of ethanol and sonicated in a sonicator for 15 min to obtain a sample that fluoresced bright blue. The crude product was purified using filter paper and filters, and the solvent was removed and further freeze-dried under vacuum to yield the final bright-blue fluorescent carbon dots. For comparison with the solvothermal method, basic tannic acid (1.0 g) was dissolved in 10 mL of ethanol, and the solution was transferred to an autoclave lined with polytetrafluoroethylene. The blue fluorescent control sample was obtained by heating in a muffle furnace at 180 °C for 6 h, cooling naturally to room temperature and filtering to remove impurities, then removing the solvent and freeze drying further under vacuum.

### 4.4. Fluorescence Detection of Metal Ions

In a typical assay, 11 metal ion solutions (Al^3+^, Ca^2+^, Fe^3+^, Mn^2+^, Zn^2+^, Mg^2+^, Na^+^, Li^+^, Cu^2+^, Ni^2+^, Ir^3+^) were prepared at a concentration of 1000 µM. To assess the selectivity of carbon dots for different metal ions, in a concentration of 1000 µM of different metal ions (e.g., Al^3+^, Ca^2+^, Fe^3+^, Mn^2+^, Zn^2+^, Mg^2+^, Na^+^, Li^+^, Cu^2+^, Ni^2+^, and Ir^3+^), 45 µL of a certain concentration of metal ions was added to 5 µL of CDs solution and incubated for 10 min at room temperature to record the fluorescence emission spectra at the optimal excitation wavelength. A series of metal ion solutions (pH 7.0) at concentrations of 0, 20, 50, 80, 100, 150, 200, 300, 400, 500, 600, 700, 800, 900 and 1000 µM were prepared in the metal ion solution (pH =7.0) and added to the carbon dot solution in the same manner, and all measurements were carried out three times. 

## Figures and Tables

**Table 1:** Elemental proportions and chemical bonds in CDs-Hydro and CDs-250-400.

	C1s	O1s	C=C/C-C	C-O	C=O
CDs-Hydro	69.1%	30.9%	97.1%	1.8%	1.1%
CDs-250	76.1%	23.9%	86.2%	9.8%	4.0%
CDs-300	81.3%	18.7%	93.0%	4.4%	2.6%
CDs-350	82.4%	17.6%	91.5%	4.6%	3.9%
CDs-400	78.9%	21.1%	90.6%	6.5%	2.9%

## Data Availability

The data presented in this study are available on request from the corresponding author.
